# Mechanisms of Fish Macrophage Antimicrobial Immunity

**DOI:** 10.3389/fimmu.2018.01105

**Published:** 2018-05-28

**Authors:** Leon Grayfer, Baris Kerimoglu, Amulya Yaparla, Jordan W. Hodgkinson, Jiasong Xie, Miodrag Belosevic

**Affiliations:** ^1^Department of Biological Sciences, George Washington University, Washington, DC, United States; ^2^Department of Biological Sciences, University of Alberta, Edmonton, AB, Canada

**Keywords:** teleost, monocyte, macrophages, antimicrobial, cytokine, respiratory burst, nitric oxide, nutrient depravation

## Abstract

Overcrowding conditions and temperatures shifts regularly manifest in large-scale infections of farmed fish, resulting in economic losses for the global aquaculture industries. Increased understanding of the functional mechanisms of fish antimicrobial host defenses is an important step forward in prevention of pathogen-induced morbidity and mortality in aquaculture setting. Like other vertebrates, macrophage-lineage cells are integral to fish immune responses and for this reason, much of the recent fish immunology research has focused on fish macrophage biology. These studies have revealed notable similarities as well as striking differences in the molecular strategies by which fish and higher vertebrates control their respective macrophage polarization and functionality. In this review, we address the current understanding of the biological mechanisms of teleost macrophage functional heterogeneity and immunity, focusing on the key cytokine regulators that control fish macrophage development and their antimicrobial armamentarium.

## Introduction

**Figure 1:**
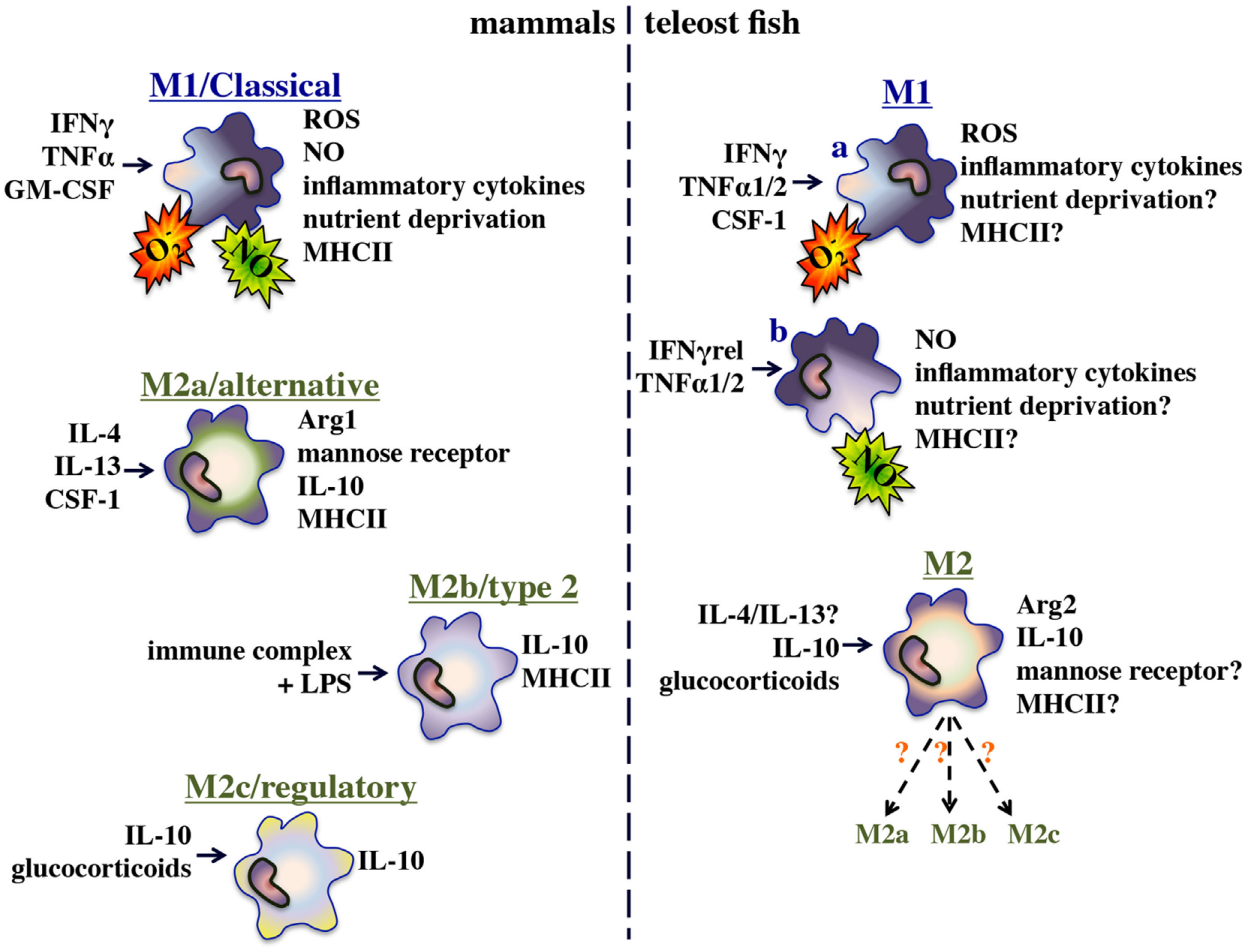
Functional polarization of mammalian and bony fish macrophages.

The immune systems of all vertebrates are integrally dependent on macrophage-lineage cells and while the ontogeny of functionally disparate macrophage subsets and lineages have been thoroughly studied in mammals (1, 2), they remain to be adequately defined in aquatic vertebrates such as teleost fish [previously reviewed by Hodgkinson et al. (3)]. In mammals, these functionally distinct macrophage subsets are framed by polarized extremes including the interferon-gamma (IFNγ) and tumor necrosis factor-alpha (TNFα) primed M1/classically activated macrophages; the interleukin-4 and/or interleukin-13-stimulated M2a/alternatively activated macrophages; the immune complexes or apoptotic cell-stimulated M2b/alternatively activated macrophages; and the interluekin-10 (IL-10), transforming growth factor-beta (TGF-β) and/or glucocorticoid (GC)-primed M2c/alternatively polarized macrophages (4). Depending on their respective stimulus-dependent polarization states, these macrophage subsets participate in either inflammatory/microbicidal or repair/wound-healing/immune suppression responses (4). While bony fish clearly possess functional analogs of these mammalian macrophage subsets (Figure 1), the molecular mechanisms governing the polarization and functionality of these respective fish macrophage populations remain to be fully defined.

The teleost fish inflammatory/M1 macrophage populations have been the best-studied and shown to rapidly kill pathogens through phagocytosis (5), production of reactive oxygen and nitrogen intermediates (6, 7), and restriction of nutrient availability (8, 9). Furthermore and akin to their mammalian counterparts, these fish M1 macrophages produce a plethora of inflammatory cytokines, chemokines, and lipid mediators (9). In a recent effort to gain insights into the alternatively polarized/M2 fish macrophages, researchers have examined functional macrophage parameters such as *arginase* gene expression and activity (10) and their expression of immunosuppressive cytokines such as *il10* and *tgf*β (11–13). Indeed, the functional analogs of the mammalian M1/M2a–c macrophage subsets appear to be present in teleosts. However, defining the regulatory mechanisms governing the polarization of these effector populations is a far more challenging goal as gene-specific and whole genome duplication events have endowed disparate fish species with unique multi-copy repertoires of those genes, which in mammals are though to dictate macrophage polarization and functionality (14).

In this review, we focus on the current understanding of the molecular mechanisms of fish macrophage antimicrobial responses to prokaryotic and eukaryotic pathogens.

## Macrophage Ontogeny

### Macrophage Sources and Fates

Until recently, tissues macrophages were believed to arise from circulating monocyte precursors in response to tissue entry and accompanying stimuli (15). However, more recent research has challenged this notion and suggests that while mammalian blood monocytes may enter into tissues and become macrophages under certain inflammatory conditions, these events are infrequent (15–17). Instead, mammalian resident tissue macrophages are now thought to be seeded during embryonic hematopoiesis and replenish resident populations locally (16–18). While the presence of self-renewing fish tissue macrophage populations requires further investigation (19), recent reports showed that fish lacking functional *c-myb* transcriptional regulator of adult hematopoiesis, nonetheless possess tissue macrophages suggest that this process may be conserved in teleosts (20).

### Teleost Monopoiesis and the Colony-Stimulating Factor-1 Receptor (CSF-1R)

The differentiation and functionality of most vertebrate macrophages are controlled by engagement of the CSF-1R, which is expressed on committed myeloid precursor cells and their derivative populations (21–23). The *csf1r* (*fms*) genes of different vertebrate species exhibit poor sequence identities, particularly in their extracellular domains (24–26). By contrast, the catalytic tyrosine kinase domains of CSF-1Rs are highly conserved (27, 28). The divergence of the extracellular portions of the CSF-1R molecules likely reflects the selective pressure onto this receptor of diverging (and in some cases multiple) ligands of these receptors, as these exhibit low amino acid sequence conservation. The mammalian, reptilian, avian, and teleost fish CSF-1Rs all branch into phylogenetically separate clades (26), presumably reflecting the many distinct aspects of macrophage functionality across these divergent species. In turn, these differences may reflect distinct functional contributions of these respective ligands and receptor systems to the macrophage ontogeny and functionality of the evolutionarily diverged vertebrate species.

### Colony-Stimulating Factor-1

Unlike birds and mammals that have a single alternatively spliced *csf1* gene (29, 30), many teleost fish species have two distinct *csf1* genes (*csf1.1* and *csf1.2*), which (for the most part) do not appear to undergo alternative splicing (25). Like its mammalian counterpart, the fish CSF-1 (CSF-1.1) also appears to be an important macrophage growth and differentiation factor (31, 32). Interestingly, while the mammalian CSF-1 is known for driving alternative/M2 macrophage differentiation (28), the cyprinid (goldfish) CSF-1.1 appears to facilitate the functional differentiation of inflammatory/M1-like macrophages with highly upregulated pro-inflammatory components (32). This is supported by the reports that a soluble goldfish CSF-1R (19) down-regulates macrophage pro-inflammatory responses by reducing available soluble CSF-1 (33, 34) (see section below). As teleosts possess multiple *csf1* genes and at least some fish species also encode two distinct *csf1r* genes (35), this suggests that teleost fish may have adopted more elaborate macrophage differentiation strategies to those seen in mammals.

### Interleukin-34 (IL-34) as Possible Sources of Macrophage Functional Heterogeneity

Inflammatory (M1) macrophages produce multiple inflammatory mediators that coordinate antimicrobial responses, while the alternatively activated (M2) macrophages secrete immunosuppressive and angiogenic compounds that control the resolution of inflammation [reviewed by Zhou et al. (4) and Hodgkinson et al. (3)]. The mammalian CSF-1 induces the differentiation/polarization of M2 macrophages (28), whereas the teleost CSF-1 elicits an M1-like macrophage phenotype (32). Notably, the IL-34 cytokine also ligates and activates the CSF-1R (36–38), regulating the development of mammalian osteoclasts (39, 40), Langerhans cells (41, 42), microglia (41), and B cell-stimulating myeloid cells (43). Recent work using the amphibian *Xenopus laevis* model indicated that frog macrophages differentiated by the *X. laevis* CSF-1 are highly susceptible to the emerging Frog Virus 3 *Ranavirus* whereas macrophages derived by IL-34 are important antiviral effectors (26, 44, 45). The antiviral roles of IL-34-derived macrophages remain to be fully elucidated in other vertebrates, and it is likely that akin to CSF-1, IL-34 likewise contributes to macrophage functional heterogeneity.

To date, there have been a limited number of studies addressing the contribution of IL-34 to the fish macrophage biology. Recent work indicates that the grouper IL-34 plays an important role in the fish immune response against *Cryptocaryon irritans* infections, as the expression of this gene was highly upregulated in the parasite-infected fish gill and skin tissues (46). This is consistent with the roles of the mammalian IL-34 in the differentiation and functionality of tissue resident macrophages and Langerhans cells (41, 42) and may reflect an evolutionarily conserved role for IL-34 in controlling the development of this macrophage-lineage cell type.

The trout *il34, csf1.1*, and *csf1.2* are differentially expressed in fish tissues and as well as in a number of trout-derived cell lines, suggesting disparate biological roles for these CSF-1R ligands (47). Notably, the trout *il34* exhibited high baseline tissue expression in which the authors attributes to a possible homeostatic role and that indeed could reflect the conserved role of this growth factor in tissue macrophage and Langerhans cell biology. Moreover, whereas stimulation of primary trout kidney macrophage cultures with a number of pathogen-associated molecular patterns (PAMPs) failed to elicit increases in *csf1.1* or *csf1.2* gene expression, these stimuli readily upregulated the expression of *il34* by these cells (47). Notably, the viral dsRNA mimic poly I:C elicited a particularly robust increase the macrophage *il34* expression, possibly reflecting a conserved role for the fish IL-34 in antiviral immunity, akin to the amphibian counterpart.

### Soluble CSF-1R

Cyprinid fish control their CSF-1 (and presumably IL-34) stimulation of macrophages by production of a soluble CSF-1 receptor (sCSF-1R) (33, 34, 48, 49). This soluble form of the receptor arises through alternative splicing, and is capable of ablating macrophage proliferation (48) and macrophage-mediated inflammatory responses (33, 49). The sCSF-1R is produced by mature macrophages, but not monocytes, in response to classical M2-polarizing stimuli such as apoptotic cells (34) and efficiently ablates an array of inflammatory events including leukocyte infiltration (34), macrophage chemotaxis, phagocytosis, production of reactive oxygen intermediates and recruitment of leukocytes (33). Moreover, sCSF-1R dampens fish macrophage chemokine and inflammatory cytokine expression, neutrophil recruitment while promoting the expression of the anti-inflammatory cytokine, interleukin-10 (49). It will be interesting to learn whether other fish besides cyprinids have adopted this strategy for controlling their macrophage inflammatory responses.

## Molecular Control of Macrophage Antimicrobial Armamentarium

### Pattern Recognition Receptors (PRRs) of Teleost Macrophages

During injury and/or infection, resident macrophages detect tissue damage and/or infiltrating pathogens by either extracellular or intracellular pattern recognition receptors (PRRs). The existence of immune PRRs was first proposed by Charles Janeway over 20 years ago (50). The known PRRs can be classified into five groups based on their structure and function: toll-like receptors (TLRs), C-type lectins, nucleotide-binding domain-leucine-rich repeat containing receptors (NLRs or NOD-like), retinoic acid inducible gene 1 (RIG1)-like receptors (RLRs), and absence in melanoma (AIM)-like receptors (ALRs) [reviewed by Hansen et al. (51)]. Neutrophils, monocytes, macrophages, dendritic cells (DCs), and specific epithelial and endothelial cells have PRRs (51).

In addition to specific recognition of distinct pathogen components (e.g., LPS, dsRNA, and flagellin), PRRs also detect tissue damage-associated molecular patterns (52–54). The human TLRs 1, 2, 4, 5, 6, and 10 are membrane bound while the TLRs 3, 7, 8, and 9 are located in endosomes (55). By contrast, the NLRs, RLRs, and ALRs are exclusively cytosolic (55).

Members of the TLR family share the intracellular toll-interleukin-1 receptor motifs (56). Initially identified in *Drosophila* spp. for controlling dorso-ventral patterning (57) and subsequently attributed to its anti-fungal properties (58), members of this family are now widely believed to be indispensable for immune recognition by most metazoans. Humans are currently known to have 10 TLRs (TLR 1–10) and mice possess 12 TLRs (51, 59). Birds also possess 10 TLRs, of which some are counterparts of the mammalian receptors (TLR 3–5, 7, and two forms of each TLR 1 and 2) (59). Some birds (TLR15, 16, and 21) are not found in higher vertebrates (60), and amphibians may have up to 20 TLRs (61). Bony fish possess 17 distinct TLRs, including some that are unique to fish, such as TLR 20–23 (62–64). Interestingly, not all fish have TLR4 and zebrafish TLR4 does not recognize LPS and negatively regulates NF-kB signaling (51, 65, 66). Additional research will be required to fully elucidate the function of TLRs in lower vertebrates, which will undoubtedly shed new light on the evolutionary history of these important innate immune receptors. The biology of fish TLRs has recently been a subject to several excellent review articles (67–70).

Other PRR families have also been identified in aquatic vertebrates. Although, gene synteny analyses identified a number of RLRs in birds and fish (71, 72), certain RLR genes are either absent or diverged beyond recognition. The evidence of functional conservation of fish RLRs exists (73, 74).

The NLRs were originally discovered in plants as R-proteins, which share nucleotide binding site and leucine rich repeat domains and can detect proteins delivered by pathogenic bacteria to trigger rapid activation of host defense (75, 76). The first identified mammalian NLR was the human NOD1 (also known as CARD4) by Bertin et al. (77) and Inohara et al. (78). The NOD1/CARD4 contained the typical NOD domain (also referred as the NACHT domain), which is a critical structural feature of NLRs (79–81), and NOD2/Card15 was identified searching for NOD1 homologs in genomic databases (82), and at present there are 23–34 NLRs known to exist in humans and mice, respectively.

There are several orthologs of mammalian NLRs as well as a unique NLR subfamily of receptors in bony fish (83). The first reported teleost NLRs were identified in zebrafish genome (84). Three subfamilies of NLRs were present in zebrafish, the first resembled mammalian NODs, the second resembled mammalian NLRPs, and the third was reported to be a unique subfamily of genes having similarities to both mammalian NOD3 and NLRPs (83). The existence of NLRs has been reported in grass carp (85), rainbow trout (86), channel catfish (87, 88), common carp (89, 90), orange-spotted grouper (91), goldfish (92), Japanese founder (93, 94), miiuy croaker (95, 96), and Japanese pufferfish (97). The results of these studies indicated the presence of inducible NLRs and that teleost NLRs shared the conserved structural domains with their mammalian counterparts. Studies on most of teleost macrophage NLRs primarily focused on the examination of gene expression induced by different immune stimuli and/or fish pathogens (87, 88, 92) and to a lesser extent on NLR signaling pathways in fish macrophages (98–102).

### The Type II Interferon System(s) of Bony Fish

The classical/M1 macrophage activation corresponds to macrophage upregulation of an array of inflammatory, microbicidal, and antigen presentation components, and is linked due to Th1-biased cytokine stimulation of these cells (103, 104). Specifically, this classical macrophage activation is thought to predominantly occur in response to the type II interferon cytokine, IFNγ, which is produced by Th1 helper cells and activated NK cells (105, 106). The induction of the mammalian M1 macrophages requires the co-stimulation of cells with IFNγ and TNFα (107). Conversely, these classically activated macrophages may be generated following macrophage activation through pathogen PRRs (108). While teleost fish have numerous PRRs (83, 109), the roles of fish PRRs (see previous section) in teleost M1 macrophage polarization remains to be fully addressed.

The mammalian IFNγ cytokine has been linked to an vast array of immunological processes, and was first identified from the supernatants of PHA-activated lymphocytes (110). In addition to its modest antiviral capacities, IFNγ appears to be particularly important to vertebrate host defenses against obligate and facultative intracellular pathogens (111–115). These include several important macrophage pathogens such as *Listeria monocytogenes* (116), *Leishmania major* (117), and *Mycobacterium* (118). This underlines the importance of this cytokine to macrophage immunity (111, 119–122).

The mammalian IFNγ binds the interferon gamma receptor 1 (IFNGR1), which results in the formation of a receptor complex composed of this ligand binding chain as well as the IFNGR2 signal propagation chain, ensuing in the downstream signaling cascade (123). The assembly of this signaling complex (IFNγ:IFNGR1:IFNGR2) activates Janus kinases (Jak)-1 and -2 (124), upon which phosphorylation activates signal transducer of activation-1 (Stat1) transcription factor (125). Under certain cellular conditions, stimulation with IFNγ may also activate Stat2 (126) albeit to a much lesser extent than Stat1. Moreover, IFNγ signaling typically results in the activation and nuclear translocation of several other transcriptional complexes including ISGF3 and Stat1-p48, composed of Stat1: Stat2: IRF-9 and Stat1: Stat1: IRF-9 (126–129). IFNγ signaling occurs in temporal phases, where the first sets of interferon gamma stimulated transcripts are seen after 30 min of the initial IFNγ receptor activation, and many of the products of these mRNAs then modulate subsequent IFNγ-related (IFNγrel) signaling events within the stimulated cell (130).

Teleost fish are widely known to possess *ifng* genes (131–135) and the functional roles attributed to the mammalian IFNγs appear to be conserved to these fish cytokine counterparts. For example, the trout IFNγ elicits the expression of a number of immune genes such as *γip10, mhcIIb*, and *stat1* (136), *c-type lectin, il1b, ifng, tap1, tapasin, irf1, ikb*, and *junb* in the monocyte/macrophage RTS11 cell line (137). Fish IFNγ enhances reactive oxygen species (ROS) production by primary kidney phagocytes of trout (136), goldfish (138), and carp (139). The goldfish IFNγ primes kidney-derived monocyte ROS responses in a concentration dependent manner (138) and akin to its mammalian counterpart (140, 141), the goldfish IFNγ synergizes with the goldfish TNFα (138) to prime the fish monocyte ROS response. The goldfish IFNγ also induces modest but significant increases in kidney macrophage nitric oxide responses, which are further enhanced by co-stimulation with TNFα2 (138). Interestingly, the carp IFNγ elicits significant NO responses in fish kidney phagocytes only in conjunction with a high dose of LPS (139). The large yellow croaker IFNγ enhances the primary kidney phagocyte respiratory burst and nitric oxide responses and upregulates the gene expression of inflammatory genes such as *tnfa, il1b, stat1*, and *irf1* in these cells (142). Likewise, the black seabream and the zebrafish IFNγs induce the expression of *jaks, stats*, and interferon-stimulated genes such as *irf1* and *mx* (143, 144).

Goldfish kidney-derived macrophages stimulated with IFNγ upregulate their expression of several inflammatory genes including *tnfa* isoforms 1&2, *il1b* isoforms 1&2, *il12* subunits p35 & p40, *ifng, il8* (CXCL-8), *ccl1*, and *viperin* (138). Carp kidney phagocytes treated with IFNγ and LPS increase their gene expression of *tnfa, il1b*, and *il12*; (subunits p35 & p40) (139). Carp IFNγ also induced the expression of a CXCL-10-like chemokine (*cxclb*) and inhibited LPS-induced expression of *cxcl8* (139). Together, it would appear that for the most part, the inflammatory roles of IFNγ such as its synergism with LPS and TNFα (see below) are conserved in teleosts.

### Functional Dichotomy of Fish Type II IFNs

In fish, Igawa et al. (132) identified two genes encode *ifng* isoforms, located next to the fish *il22* and *il26* genes, that have the exon/intron organization of *ifng* genes of other vertebrates and possess the IFNγ signature motif ([IV]-Q-X-[KQ]-A-X_2_-E-[LF]-X_2_-[IV]). These two *ifng* sequences were initially coined IFNγ1 and IFNγ2 but following a reevaluation of vertebrate *ifng* genes and because the fish IFNγ2 possesses the hallmark features of the mammalian IFNγs, it was renamed as simply IFNγ (145). Since IFNγ1 appears to be structurally related to the mammalian IFNγ, but is missing a nuclear localization signal (NLS) motif, it has been coined as IFNγrel. The presence of multiple *ifng* isoforms have now been confirmed in siluriformes and other cypriniformes, including the identification of *ifngrel* and *ifng* in catfish (133), common carp (146), zebrafish (147), and the goldfish (138).

The siluriform IFNγrel proteins have not been functionally characterized. However, the cyprinid IFNγrels have been examined in some detail across several species. For example, freshly laid zebrafish eggs possess *ifngrel* transcripts, indicating maternal supply of these mRNAs (147). Also, while the gene expression of the zebrafish *ifng* is not detected until much later in development, the mRNA levels of IFNγrel continue to increase during the embryonic zebrafish development (147). Moreover, injection of zebrafish embryos with mRNAs encoding IFNγ or IFNγrel results in increased expression of genes typically activated by the mammalian IFNγ (147). Notably, morpholino knock-down of either *ifng* or *ifngrel* resulted in compromised *Yersinia ruckeri-*infected zebrafish embryo survival while the combined knock-down of both cytokines further decreased embryo survival (147), suggesting that IFNγ or IFNγrel confers at least partially non-overlapping immune roles.

The goldfish IFNγ and IFNγrel appear to confer distinct effects on macrophages (138, 148). For example, while IFNγ stimulation of goldfish monocytes results in long-lasting ROS priming, IFNγrel elicits a short-lived priming effect on these cells, followed by complete monocyte unresponsiveness to ROS priming by other inflammatory cytokines (IFNγ or TNFα2). Moreover, the goldfish IFNγ only modestly enhances fish monocyte/macrophage phagocytosis and nitric oxide responses (138, 148). By stark contrast, IFNγrel induced significantly greater phagocytosis, iNOS (isoforms A and B) gene expression, and nitric oxide production in goldfish monocytes and macrophages. Interestingly, these goldfish type II IFNs also elicit the expression of distinct immune genes in goldfish monocytes. Both recombinant cytokines induce goldfish monocyte Stat1 phosphorylation, however, nuclear translocation of Stat1 was only seen in cells treated with IFNγ, but not with IFNγrel. This was confirmed by more recent report, indicating that the zebrafish IFNγ and IFNγrel utilize distinct signaling pathways (143). It is interesting that while the recombinant ginbuna crucian carp IFNγ forms a dimer in solution, the recombinant IFNγrel appears to be monomeric (149, 150), akin to the functional forms of type I rather than type II IFNs. Moreover, an additional isoform of the ginbuna carp IFNγrel has been identified and shown to possess a functional NLS, which contrasts the other fish IFNγrel proteins (150). With the growing evidence indicating functional dichotomies of the cyprinid type II IFNs, it will be interesting to learn the roles of these distinct macrophage-activating factors in their target cells’ antimicrobial responses to different fish pathogens.

### Fish Type II IFN Receptors

While the bony fish type II IFN ligands have become a subject of active research, the functional roles of the type II IFN receptors remain to be clearly defined. The trout IFNGR1 and IFNGR2 chains were initially identified and shown to exhibit conserved gene synteny across vertebrates (151). All fish IFNGR1 sequences have Jak1 and Stat1 binding sites, that are also required for functional mammalian IFNγ (152–154), and the expression of the IFNGR2 chain appears to be essential to the trout IFNγ-induced signaling (151).

The fish IFNγ and IFNγrel cytokines structural, functional, and intracellular signaling differences were thought to reflect the presence of distinct IFNγ receptors, dedicated to these respective moieties. As predicted, gene synteny analyses of the vertebrate *ifngr1* genes (encoding the ligand binding chain), revealed two distinct zebrafish *ifngr1* genes, located on distinct chromosomes (155). The presence of corresponding *ifngr* isoforms was confirmed in goldfish, and by means of *in vitro* recombinant protein binding studies, we demonstrated that IFNγrel (IFNγ1) and IFNγ each bound to their own cognate IFNγ receptor chains, the IFNGR1-1 and IFNGR1-2, respectively. Morpholino knock-down of the zebrafish *ifngr1-1, ifngr1-2*, or *ifngr2* (signal propagation chain) abolished the fish IFNγ function (156). Notably, only the knock-down of *ifngr1-1*, but not *ifngr1-2* or *ifngr2*, abrogated IFNγrel stimulation, suggesting that zebrafish IFNγ signals through a heterodimer (IFNGR1-1 and IFNGR1-2) and a IFNGR2 homodimer whereas the IFNγrel binds to homo-dimeric IFNGR1-1 and a distinct unknown receptor 2 chain. The discrepancy between these finds and our studies, which indicated IFNγ-IFNGR1-2 but not IFNγ-IFNGR1-1 interactions, could be explained in several ways. Aside from the possible species-specific differences, it may be that IFNGR1-1 binds IFNγ with lower affinity, explaining our inability top detect this interaction *in vitro* by western blot. Conversely, the presence of *ifngrel* mRNA in fresh zebrafish embryos (147), suggests that this cytokine may plays roles during zebrafish (and presumably other cyprinid fish) development. If is the case, morpholino knock down of its cognate receptor encoding gene, *ifngr1-1* may manifest in reduced IFNγ function, as an indirect consequence of the abrogated IFNγrel-mediated immune development rather than through direct IFNγ–IFNGR1-1 interactions.

It is notable that using HeLa cells transfected with the ginbuna carp *ifngr1-1* and *ifngr1-2* encoding plasmids, it was shown that the carp IFNγ isoform 1 exclusively signals through the IFNGR1-2 whereas the IFNγ isoform 2 signals through the IFNGR1-1 (149). It is well established that both the mammalian and fish IFNγ signaling requires IFNGR2 chains (123, 156) while the fish Jak and Stat proteins have significantly diverged from (and are present in multiple forms as compared to) the mammalian counterparts (157).

While all other vertebrates examined to date encode individual type II IFNs and IFNGR1 genes, it is intriguing that certain fish possess two distinct IFN gamma-receptor binding chains (IFNGR1-1 and IFNGR1-2) as well as multiple type II IFNs (148, 149, 156). This suggests that these fish have adopted very unique strategies surrounding their principal M1 macrophage-activating cytokine system(s) and it will be exciting to learn what are the functional consequences of these differences.

### Teleost TNFα

The mammalian TNFα is involved in a broad array of immunological roles (158–160). The name of this cytokine stems from its discovery in tumoricidal sera of Bacillus Calmette-Guerin-primed, endotoxin-treated mice (161). During vertebrate inflammatory response, TNFα promotes the chemotaxis of neutrophils and monocytes/macrophages to the sites of inflammation (162, 163), enhance macrophage phagocytosis (164–166), primes reactive oxygen and reactive nitrogen responses (167, 168), facilitates the chemotaxis of fibroblasts (169) and the release of platelet activating factors (170–172). Mammalian TNFα confers its immune effects either as a 17 kDa soluble protein or a 26 kDa type II trans-membrane protein (173–175) and most effects are induced after binding of homotrimerized TNFα to either the TNF-R1 or TNF-R2 (176, 177).

Tumor necrosis factor-alpha orthologs, possessing the TNF family signature [LV]-x-[LIVM]-x_3_-G-[LIVMF]-Y-[LIMVMFY]_2_-x_2_-[QEKHL] have been identified in several teleosts (178), underlining the evolutionary conservation of this cytokine. Like its mammalian counterpart, the teleost fish TNFα is a reliable marker of fish M1 macrophages (179, 180). Most fish species possess multiple TNFα isoforms (178, 181–188). These TNFα isoforms confer pro-inflammatory effects such as enhancing inflammatory gene expression, macrophage chemotaxis and phagocytosis, and eliciting phagocyte reactive oxygen and nitrogen intermediate production (183–185, 189–194). The *in vivo* roles of TNFα during fish inflammatory and M1 macrophage immune responses have also been confirmed in zebrafish (179), sole (195) and trout (196, 197).

### Teleost TNFα Receptors

Bobe and Goetz (198) were first to report the presence of a death domain-containing TNF receptor in zebrafish and coined this gene the ovarian TNF receptor (*otr*), while putative zebrafish *tnfr1* and *tnfr2* gene sequences were deposited to GenBank, with the zebrafish *tnfr1* sharing high sequence identity with *otr*. We identified the goldfish *tnfr1* and *tnfr2* cDNAs (199) and showed that the putative amino acid sequences of these goldfish receptors share many conserved regions with their respective mammalian counterparts. Goldfish TNF-R1 has a death domain with a conserved motif (W/E)-X_31_-L-X_2_-W-X_12_-L-X_3_-L and six residues that are essential to TNF-R1-mediated cytotoxicity (200).

Our *in vitro* binding studies using recombinant version of the respective goldfish proteins indicate that both goldfish TNFα1 and TNFα2 bind either TNF-R1 or TNF-R2 (199). Notably, recombinant sea bream TNFα (191), and the goldfish recombinant TNFα1, TNFα2, TNF-R1, and TNF-R2 all adopt homo-dimeric conformations and associate as dimers as opposed to the trimeric confirmations seen in the mammalian TNF ligands and receptors (199). Similarly, the grass carp TNFα ligand and TNF-R1 also associate as dimers (201). Interestingly, dimerized forms of the mammalian TNF-R1 have been observed (202–204) while the mammalian TNF receptor superfamily member, neurotrophin receptor (p75/NTR), is structurally similar to the teleost TNF-R1 and binds to the NTR ligand as a dimer (205, 206).

By studying the TNF systems of teleost fish, we may garner greater insights into the evolutionary origins of these important and evolutionarily conserved cytokines and receptors. Indeed the importance of the teleost TNFα proteins to their immune defenses is underlined by the fact that a number of diverse viral fish pathogens encode decoy TNF receptors (207–210).

### Macrophages and Acute Phase Proteins (APPs) of Bony Fish

During inflammation, activated macrophages secrete cytokines and oxidative radicals that modulate the production of APPs by hepatic cells [reviewed by Gruys et al. (211)]. These APPs opsonize pathogens, activate complement, neutralize enzymes, and scavenge free hemoglobin and radicals.

Acute phase proteins rapidly increase in the blood early after exposure to pathogens or during early inflammatory response. For example, blood levels of C-reactive protein (CRP) may increase as much as 1000-fold and 50% increases in complement proteins and ceruloplasmin (*Cp*) have been observed. The activation of hepatocytes also results in decreased levels of serum transferrin, cortisol-binding globulin, zinc, iron, albumin, and retinol, as well as reduction of free hormones in the blood (212).

While viral infections induce modest acute phase responses (213), bacterial infections elicit potent production of these soluble mediators (211, 214–216). Upon recognition of LPS, monocytes and macrophages also produce gratuitous amounts of pro-inflammatory cytokines (214, 216–219). The termination of APP production is controlled by pro-inflammatory cytokines secreted by macrophages (220, 221).

Bony fish have fully functional repertoires of APPs, which are shared with their mammalian counterparts, as well as additional APPs that are unique to teleosts. The serum-CRP levels of salmonids have been used as indicators of stress in response to xenobiotics (222–224), and protozoan infections (225). The infection of goldfish with *Trypanosoma carassii* increased expression of *Cp, crp*, and *serum amyloid A* (*saa*), in the liver, particularly during the early phases of the infection (first 14 days of infection) (225). Serum amyloid-A (SAA) and a serum amyloid P-CRP-like pentraxin proteins have also been identified in salmonids (226), and goldfish (227). *Aeromonas salmonicida* infection of salmon also induced increased levels of SAA protein (226), while goldfish recombinant SAA was shown to induce increased gene expression of *il12p40* and *il1b*, and was chemotactic to primary goldfish macrophages (227). It has also been demonstrated that similar to mammals, trout CRP was capable of activating complement (228).

The salmon *saa* was shown to be upregulated in hepatocytes after their exposure to supernatants from LPS-activated macrophages, or recombinant TNFα, IL-1β, or IL-6 (229). Interestingly, while LPS stimulations increased the expression of the fish pentraxin, *A. salmonicida* infections downregulated the expression of this gene, suggesting that pentraxin may be a “negative” APP (226, 230).

A selective subtractive hybridization (SSH) study of hepatic transcripts in unchallenged and bacterially challenged trout confirmed that a fully functional, broad-repertoire acute phase response exists in teleosts (378). Furthermore, after exposure to distinct pathogens, trout produce overlapping but partially distinct profiles of APPs (231). Catfish also have a well-developed acute phase response following bacterial infections leading to a 50-fold increase in the expression of some of the genes that encode APPs (232). In zebrafish, SSH analysis revealed that zebrafish infected with *A. salmonicida* and *Staphylococcus aureus* possess overlapping as well as unique APPs to those reported in mammals (233).

### Macrophages and Complement

During a pathogen insult or PAMPs-induced inflammatory responses, there is a significant increase in blood complement levels [reviewed by Mastellos et al. (234) and Markiewski and Lambris (235)]. Most of the mammalian complement components exist in bony fish [reviewed by Nonaka (236)]. When compared with mammals, birds, and amphibians; teleosts have a full set of complement genes with the exception of Factor D, and the absence of MASP-1 and MASP-2 (236). Thus bony fish have multiple forms of several complement components including C3 and C5 proteins (237–241).

Fish complement components have similar pro-inflammatory roles akin to those of mammals. The anphylatoxin, C5a, has chemo-attractive activity (237, 240) and trout C3a enhances fish leukocyte phagocytosis (238, 241). In addition trout C3a, C4a, and C5a has been shown to be chemo-attractive to head kidney phagocytes and PBLs, and enhance phagocytosis of kidney leukocytes (242). The teleost complement biology has been fully addressed in a review by Sunyer et al. (243).

## Antimicrobial Roles of Teleost M1 Macrophages

### Phagocytosis

Phagocytosis is the primordial defense mechanism of all metazoan organisms. During the inflammatory response monocytes/macrophages and neutrophils, undergo phagocytosis mediated *via* phagocytic receptors or hydrophobic interactions of the phagocyte membrane and the target particles. Once activated, phagocytes release numerous preformed or newly synthesized inflammatory mediators, and are equipped with an armamentarium of antimicrobial responses primarily focused on the pathogens enclosed in the phagolysosomes. Potent antimicrobial compounds generated by activated phagocytes include degradative enzymes (proteases, nucleases, phosphatases, and lipases) and antimicrobial peptides (basic proteins and neutrophilic peptides), which mediate the destruction of phagocytosed pathogens (244–249).

### Respiratory Burst Response

**Figure 2:**
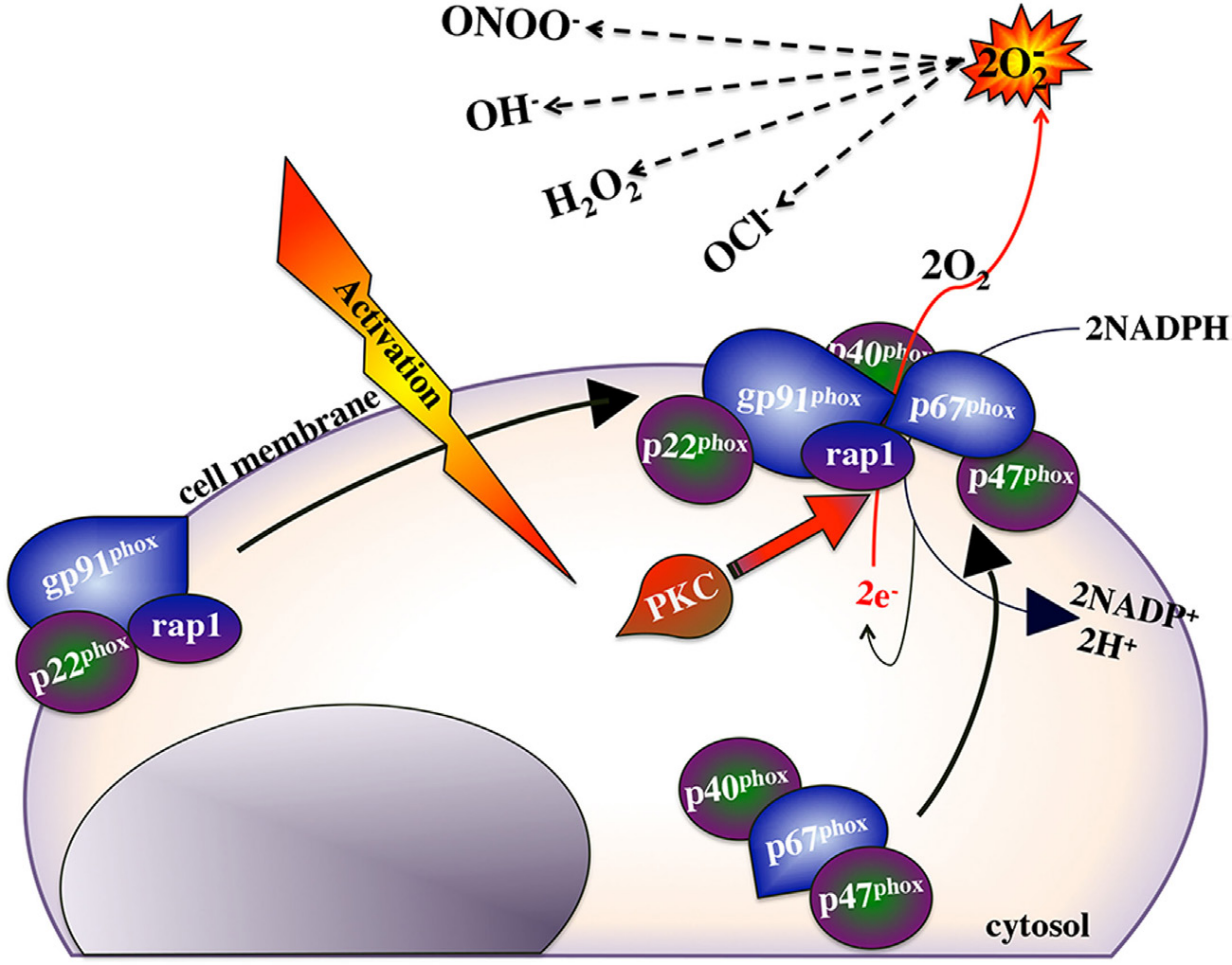
NADPH oxidase and the reactive oxygen response.

Macrophage ROS response is a hallmark of these cells’ antimicrobial armamentarium and the efficacy of this response often reflects on the ability of macrophages to destroy internalized microorganisms. This response culminates from the assembly of a multicomponent enzymatic complex, the nicotinamide adenine dinucleotide phosphate (NADPH, Figure 2) oxidase on the plasma and phagosome membranes, resulting in the transfer of electrons from NADPH to molecular oxygen and thus the production of a superoxide anion (250). In turn, the generated superoxide anions may be converted into other antimicrobial ROS such as hydrogen peroxide (H_2_O_2_), hydroxyl radical (OH^⋅^), and hypercholorus acid (251, 252). The NADPH oxidase complex has six interactive subunits including the cytosolic phagosome oxidases (p40phox, p47phox, and p67phox), and a guanosine triphosphatase Rac 1 or Rac 2, which are mobilized to the gp91phox and p22phox subunits that are located in the plasma membrane (253–258). All of these NADPH oxidase components have been identified in teleosts and fish macrophage ROS responses has been well documented in contexts of PAMP stimulation (259–262), antimicrobial responses (263–265), and recombinant cytokines stimulation such as with TNFα (183, 184, 266), IFNγ (136, 138, 148), and CSF-1 (32).

### Tryptophan Degradation

**Figure 3:**
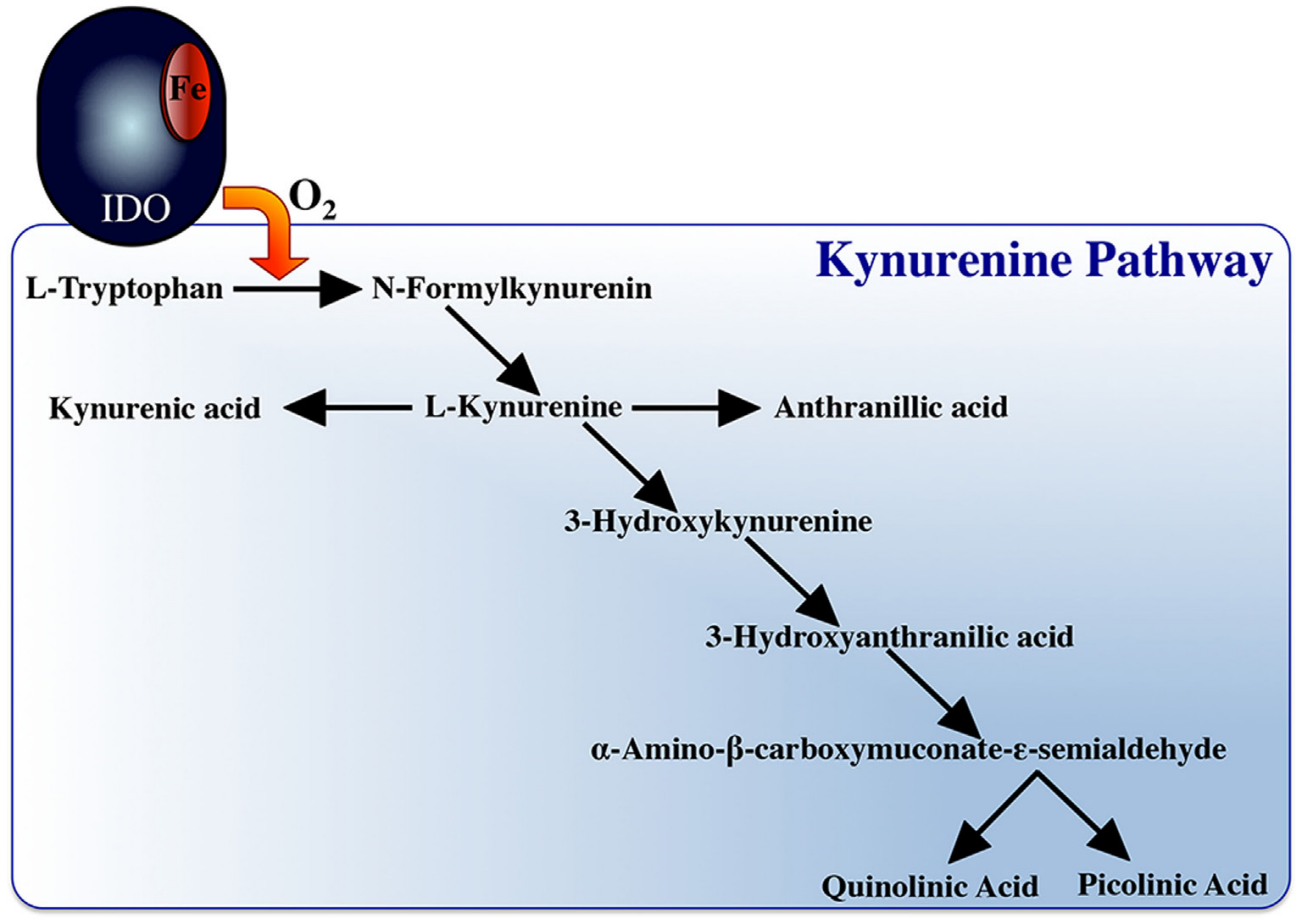
Indoleamine 2, 3 dioxygenase and the tryptophan degradation pathway.

Another hallmark of M1 macrophages is their capacity to deplete local tryptophan levels through their upregulated expression of the indoleamine 2,3-dioxygenase (IDO) enzyme (267) (Figure 1), which catalyzes this process (268). IDO-mediated tryptophan degradation is closely linked to macrophage antimicrobial responses but also to their immunoregulatory functions, as this tryptophan degradation results in the production of metabolites such as kynurenins ((269), Figure 3), which may inhibit T cell proliferation. IFNγ-stimulation of macrophages has been closely linked to inducing the mammalian macrophage IDO response (270–273).

The teleost IDO orthologs (renamed proto-IDOs) are less effective at tryptophan degradation than the mammalian IDOs (274), bringing to question whether these fish enzymes have distinct substrates. Interestingly, *Mycobacterium marinum*-challenged goldfish macrophages upregulate their *proto-ido* gene expression (275), suggesting a possible M1 role for this fish enzyme.

### Nitric Oxide Response

**Figure 4:**
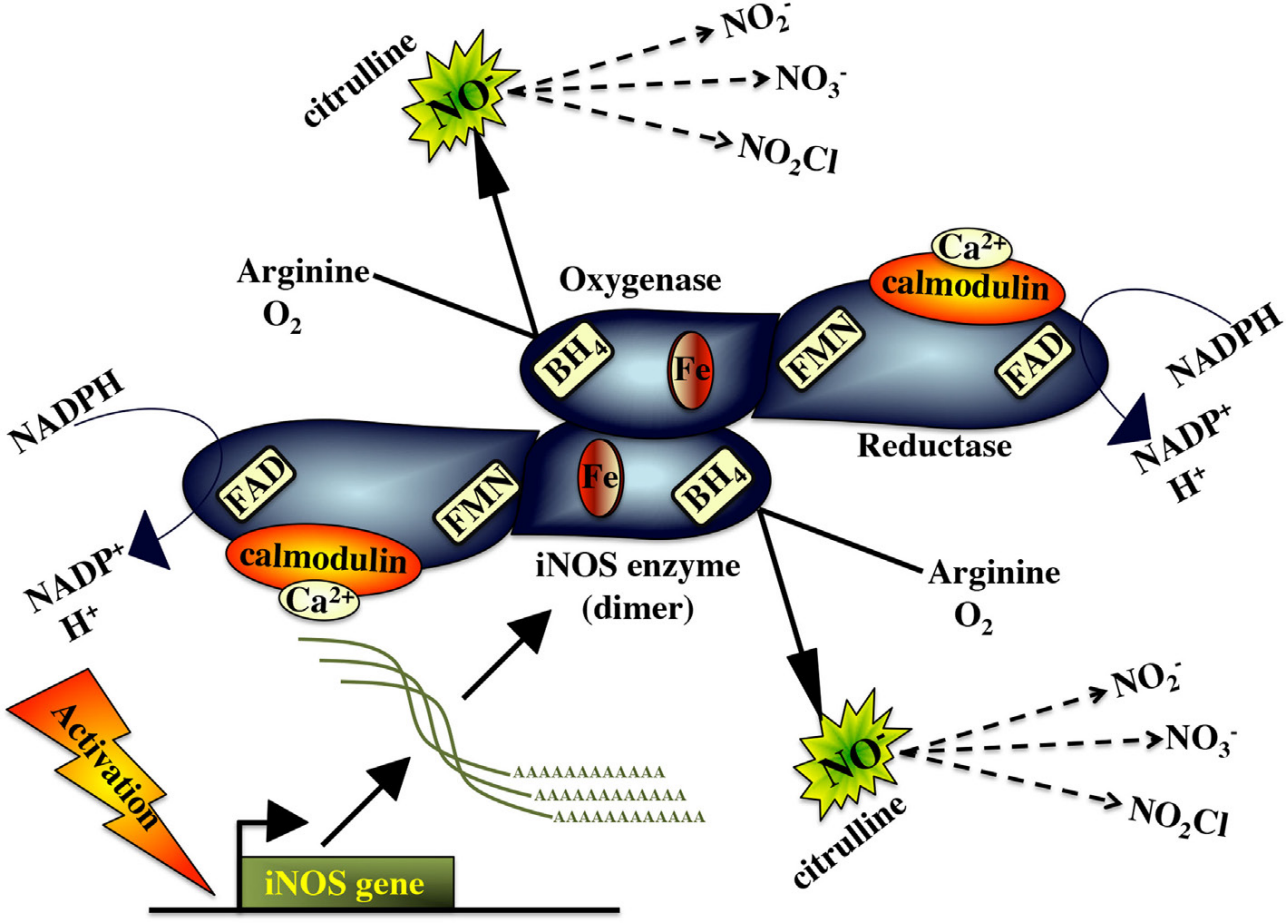
Inducible nitric oxide synthase and the nitric oxide response.

Classically activated M1 macrophages possess high levels of the inducible nitric oxide synthase enzyme (iNOS/NOS2), which catalyzes the conversion of L-arginine to L-citrulline, resulting in the production of nitric oxide (NO) (276) (Figure 4). As such, iNOS expression serves as a marker of M1 macrophage activation, which may be enhanced by macrophage stimulation with IFNγ, TNFα, and/or microbial compounds (e.g., LPS) (106). The parallel production of superoxide and NO can also result in the formation of peroxynitrite (ONOO^-^), which is a potent antiparasitic/antimicrobial agent (277). The immune mechanism governing the teleost macrophage inducible nitric oxide (NO) appears to be well conserved to those described in mammals.

Akin to its mammalian counterpart, the fish iNOS has putative binding sites for heme, calmodulin, flavine mononucleotide, flavine adenine dinucleotide tetrahydrobiopterin, and NADPH, indicating that this is a highly conserved enzyme (278). The fish macrophage iNOS gene is induced by antimicrobial and inflammatory stimuli such as PAMPs/pathogen recognition (10, 11, 278, 279), pro-inflammatory cytokines (138, 139, 183, 187) and cleaved transferrin products (280, 281). In turn, effective fish macrophage nitric oxide production is integral to fish antimicrobial immunity to a range of pathogens (282–287).

### Sequential Induction of Macrophage Antimicrobial Responses

While mammalian macrophages are thought to be able to undergo simultaneous ROI and NO responses (288), there are several reports suggesting that teleost (primarily cyprinid) fish mount and sequentially deactivate their antimicrobial responses (138, 184, 260, 289, 290). We are aware of only one report describing sequential mammalian macrophage production of ROS followed by NO (291). However, the interdependence of the respective mammalian macrophage respiratory burst, tryptophan degradation, and nitric oxide responses suggest that sequential regulation of macrophage antimicrobial responses is not a strategy that is unique to teleosts and may be a predetermined fail-safe component of all vertebrate macrophage antimicrobial responses.

**Figure 5:**
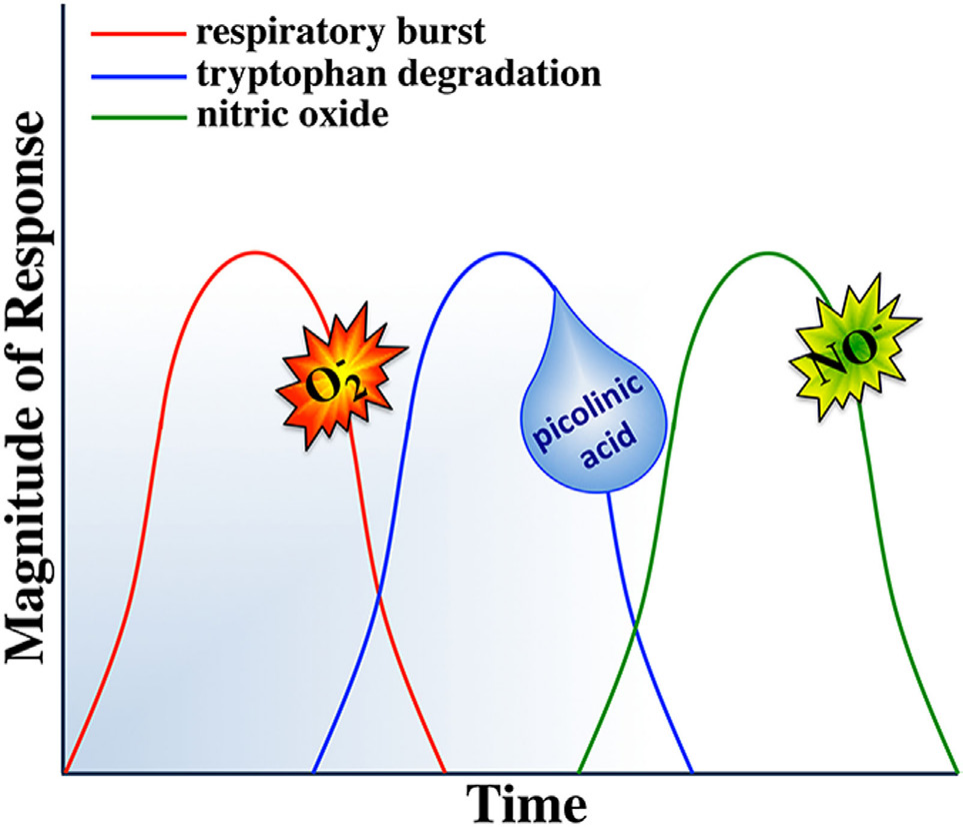
Sequential activation of macrophage responses.

The respiratory burst and nitric oxide responses are thought of, as two independent macrophage microbicidal mechanisms, where in the induction of one does not depend on the induction of the other (288). However, both responses may be linked to tryptophan degradation. IDO activation requires reduction of its ferric (Fe^3+^) heme to ferrous (Fe^2+^) heme and there has been some contention regarding the source(s) of electrons used toward this reduction of the IDO heme (292, 293). Interestingly, a prevailing theory suggests that the superoxide anion, derived from the respiratory burst response, is in turn shunted into this enzymatic pathway, serving as this electron source (270, 273, 294, 295). It is interesting to consider that the sequential induction of the respiratory burst response before tryptophan degradation would ensure sufficient quantities of superoxide as a substrate for IDO activity and in turn would repurpose any remaining superoxide anions that had not reacted with the pathogen, thereby also minimizing bystander host cell damage. This notion is supported by the fact that the metabolites from tryptophan degradation are potent scavengers of ROS (296, 297). This in mind, simultaneous induction of macrophage tryptophan degradation and the respiratory burst response would thus be an overall inefficient microbicidal strategy, as the ROS would be actively scavenged by tryptophan catabolites. Thus, sequentially mounting these responses (Figure 5) would maximize the targeted effects of the respective responses.

Notably, macrophage tryptophan degradation appears to also be coupled to production of nitric oxide. Picolinic acid, a catabolite of tryptophan degradation (Figure 3), synergizes with IFNγ to induce nitric oxide production in murine macrophages (298–302). Picolinic acid exerts this nitric oxide inducing potential *via* a hypoxic responsive element located in the 5’ flanking region of the murine iNOS gene, while mutation or deletion of this promoter sequence impairs picolinic acid-induced gene transcription of iNOS without affecting induction of nitric oxide synthase by LPS (300). Thus, we propose that staggering the kinetics of macrophage tryptophan degradation and nitric oxide production would ensure sufficient quantities of picolinic acid toward the synergistic induction of nitric oxide. In turn, if the respiratory burst response was concomitantly induced with nitric oxide production, then picolinic acid could not exert its nitric oxide inducing effects, as the respiratory burst creates an extremely hyperoxic microenvironment. We thus suggest that the induction of tryptophan degradation before nitric oxide production would facilitate the establishment of a hypoxic microenvironment due to the tryptophan catabolites actively scavenging reactive oxygen intermediates, permitting picolinic acid to augment nitric oxide production by macrophages.

Nitric oxide appears to be the terminal microbicidal response of vertebrate macrophages. In addition to its potent killing effects, nitric oxide is a deactivator of specific enzymes involved in macrophage cytotoxic reactions. Interestingly, NO inhibits both protein kinase C (needed for initiating the ROI response; Figure 2) and IDO enzymes involved in the activation of the respiratory burst and tryptophan degradation, respectively (303, 304). Moreover, nitric oxide acts as a negative feedback inhibitor of its own synthesis (305, 306). Therefore, simultaneous induction of nitric oxide, respiratory burst and tryptophan degradation responses would antagonize PKC and thus NADPH oxidase activation (Figure 2) and the IDO enzyme. By sequentially inducing the nitric oxide response, subsequent to the respiratory burst and tryptophan degradation responses would ensure that each of these responses would be maximally induced and terminated in a timely manner, thus maximizing these respective antimicrobial responses and minimizing off-target effects of each response.

Based on the above and as outlined in Figure 5, we propose that such sequential induction and deactivation of macrophage antimicrobial responses may represent an important and presently poorly explored component of macrophage defenses. As activated macrophages are highly cytotoxic, the interdependence and temporal segregation of their individual microbicidal responses likely represents an inherent way to minimize host cell damage and concomitantly to maximize pathogen elimination. For example, pathogenic microorganisms that are susceptible to ROI are rapidly killed upon phagocytosis by activated macrophages while those pathogens that are resistant to oxidative burst, are often susceptible to subsequent nutrient deprivation and/or antimicrobial attacks. Indeed, ablating the macrophage respiratory burst response while shunting the produced superoxide anion into tryptophan degradation and the subsequent utilization of the picolinic acid from this response toward NO production (Figure 5) would maximize the effectiveness of each respective response. This would allow macrophages to divert and target their metabolic energy into distinct, targeted and timely antimicrobial assaults.

The proposed model shown in Figure 5 does not define macrophage activation in the context of a given individual macrophage, and indeed individual macrophages do not necessarily have to cycle through all of the above responses. Moreover, while much contention remains regarding the functionality of dipartite mammalian macrophage subsets, teleosts clearly possess macrophage sub-populations exhibiting dramatically different kinetics of activation and distinct antimicrobial capacities (9, 148, 184, 290). Notably, cyprinid kidney-derived monocyte-like cultures are considerably more proficient producers of ROS whereas the maturation of these cultures into predominantly macrophage-like cells coincides with their loss of respiratory burst capacities and a concomitant gain of significantly more robust NO responses (184). Presumably, sub-populations of macrophages with distinct antimicrobial potentials coordinate the sequential induction of macrophage antimicrobial responses *in vivo*.

It is unclear why despite considerably more rigorous investigation of the mammalian macrophage, there is more evidence of sequential macrophage antimicrobial responses in teleosts. The central M1/classical activation strategies of mammals and teleosts are best framed by their respective functional polarization by IFNγ. As described above, mammalian species possess single IFNγ molecules that are important for the activation of M1 macrophage ROI and NO responses (Figure 1). Intriguingly, many teleost fish possess multiple distinct IFNγ proteins, some of which appear to be potent elicitors of the macrophage ROS, but not NO responses whereas others elicit robust NO production but meager ROIs (148). Thus, we argue that these fish species may have evolved to generate multiple distinct M1 macrophage populations, here denoted as M1a and M1b (Figure 1). As an extension of this notion, we argued that fish may have evolved this relatively elaborate classical macrophage activation strategy in order to better coordinate, and when needed, segregate their respective macrophage antimicrobial responses.

## Activation of Alternative/M2 Teleost Macrophages

### Interleukin-4/13

M2 macrophages have ‘anti-inflammatory’, or ‘pro-healing’ phenotypes and the most extensively characterized M2-polarizing agents (sometimes called M2a) are the IL-4 and IL-13 cytokines (Figure 1), which are typically produced by Th2 cells, eosinophils, basophils, NK-T cells and certain macrophages subsets (307). IL-4 binds to the IL-4 receptor-alpha and either the IL-4 receptor-gamma or the IL-13 receptor-alpha1 chains, culminating in Jak1, Jak3, and Stat6 downstream signaling (104). IL-13 also ligates the IL-13 receptor-alpha2 chain (104). Either of these M2 stimuli result in increased of expression/production of a number of hallmark M2 macrophage components including transglutaminase 2, prostaglandin-endoperoxide synthase, transcription factors IRF4, macrophage mannose receptor, and suppressor of cytokine signaling 1 (SOCS1), all of which are present in fish but await to be functionally linked to teleost M2 macrophages (308–313).

Teleost possess IL-4/13A and IL-4/13B genes with sequence homology to both the mammalian IL-4 and IL-13 cytokines (314). These fish cytokines are thought to have arisen from genome/gene duplication events, and are present in distinct copies in different fish species (315). Paralogs of IL-4Rα, IL-13Rα1 and IL-13Rα2 have also been identified in teleosts (316, 317), while the recombinant fish IL-4/13A induces B and T cell expansion in an IL-13Rα-dependent manner (318, 319), suggesting that the roles of these fish cytokines possess the immune roles of their mammalian counterparts. The fish IL-4/13A and IL-4/13B are thought to play the M2/anti-inflammatory roles attributed to the mammalian IL-4 and IL-13 (320) and the trout, seabass, grass carp and goldfish recombinant IL-4/13A and IL-4/13B possess many of these anti-inflammatory roles including the upregulation of immunosuppressive genes (TGF-β, IL-10, SAP1, and SOCS3); dampening of pro-inflammatory cytokine gene expression (TNFα, IL-1β, and IFNγ); as well as elevating macrophage/kidney phagocyte arginase gene expression and arginase activity (321–324). Notably, a true Th2 locus has been identified in spotted gar, consisting of RAD50, IL-4/13 and IL-3/IL-5/GM-CSF (IL-5) (325) while the constitutively high expression of trout and salmon IL4/13A in the thymus, skin and gill tissues have been attributed to immunological tolerance and thus a Th2-like response (320).

### Arginase

The enhanced capacity to metabolize L-arginine marks an important paradigm between M1 and M2 macrophages and underlines the M2 macrophage. This is intuitive, as M1 macrophage armamentarium is known for its elevated iNOS enzyme, which converts L-arginine to L-citrulline and NO. By contrast, the M2 macrophage arginase enzyme converts L-arginine to L-ornithine and urea (326, 327). The tissue repair capacities of these M2 macrophages in turn reflect their production of L-ornithine, which serves as a precursor for polyamines and proline components of collagen, during tissue repair (328). Notably, the products of these iNOS and arginase enzymatic pathways serve as reciprocal inhibitors of these antagonistic enzymes, promoting the respective M2 or M1 macrophage phenotypes (329).

Mammals possess two arginase isoforms, of which the macrophage gene expression of arginase-1 is induced by IL-4 and IL-13 (330). By contrast, macrophage *arginase-2* gene expression is upregulated by IL-10 and LPS (331). Fish possess both *arginase*-1 and *arginase-2* (332) and like mammals the fish M1/M2 paradigm is outlined by respectively elevated macrophage *inos* and *arginase* genes (10, 11, 279). By contrast to the mammalian M2 macrophages, carp alternative macrophage activation results in the induction of *arginase-2* rather than *arginase-1* expression (10). The facets of fish macrophage M2 polarization and the roles of arginase-2 to in this process have been thoroughly reviewed (190, 333).

### GCs and Interleukin-10

Glucocorticoids and IL-10 stimulation of macrophages culminates in a unique regulatory macrophage phenotype, otherwise known as M2c. GCs diffuse across plasma membranes, resulting in alterations to the expression of a plethora of immune-related genes, which results in these M2c macrophage transcriptional profiles that are distinct from those seen in IL-4/IL-13-stimulated macrophages (334, 335). These M2c macrophage transcriptional changes include decreased inflammatory cytokine gene expression and dampening of ROS production. In line with the immunosuppressive nature of GCs, cortisol increases fish susceptibility to diseases (335, 336) and inhibits fish macrophage NO production (337). Moreover, the simultaneously of fish macrophage cell lines with combined pro-inflammatory stimuli and cortisol results in elevated *il10* gene expression (13), indicating that the cortisol treatment overrides the inflammatory stimuli.

The mammalian IL-10 cytokine signals through a receptor complex composed of IL-10 receptors 1 (IL-10R1) and 2 (IL-10R2), leading to downstream STAT3 activation, which results in decreased gene expression of pro-inflammatory cytokines (338). Macrophage IL-10 production may be elicited by TLR agonists, GCs, and C-type lectins (307). Fish IL-10R1 has been identified in several cyprinids (339, 340), while the IL-10R2 has been reported in salmonids (341). Consistent with the mammalian counterpart, the goldfish recombinant IL-10 down-regulates macrophage ROS responses and inflammatory gene expression (275).

## The Macrophage Bridge Between the Innate and Adaptive Immunity

In addition to their roles in early antimicrobial responses, macrophage-lineage cells are crucial to bridging the innate and adaptive arms of the vertebrate immune response. To this end, mammalian macrophages present intracellular pathogen-derived antigens to conventional CD8^+^ cytotoxic T cells *via* the MHC I pathway (342); extracellular antigens to CD8^+^ T cells in the context of MHCI by means of antigen cross-presentation (343) and extracellular antigens to conventional CD4^+^ T helper cells by means of MHCII complex (344). In addition, myeloid cells may present non-protein antigens to unconventional lymphocytes, such as lipid antigens in the context of non-classical MHCI (CD1) to invariant T cells and NK-T cells (345). Moreover, macrophages readily clean up antibody-opsonized pathogens through Fc-receptor-mediated phagocytosis (346). The molecular mechanisms by which teleost fish macrophages bridge the innate and adaptive arms of their respective immune responses are by far the most poorly understood.

### Teleost Antigen Presentation

The fish (salmonid) MHCI peptide-loading complex appears to be fundamentally and functionally similar to that of mammals and the macrophage-like (RTS11) trout cell line has been demonstrated to assemble this antigen presentation complex (347–349). Moreover, trout appear to possess an alternatively spliced variant of MHCI loading glycoprotein, tapasin (349), which is believed to serve as additional regulatory mechanisms in the fish MHCI antigen presentation pathway. While some fish species such as medaka, sharks (350) and zebrafish (351) possess considerable polymorphism within their respective MHCI loci, other species such as Atlantic salmon do not have significant polymorphisms within their classical MHC I antigen processing genes (352). Interestingly, some of the other salmon MHC I assembly and antigen processing genes have been retained as functional duplicates (352). It is thought that these duplicated gene originated from the second vertebrate genome duplication event and are now providing various fish (and some tetrapods such as frogs and birds) with the potential of several different peptide-loading complexes (352).

Several teleost lineages have independently lost key components associated with mammalian antigen presentation and immunological memory including MHCII and CD4 (353–355), although these species exhibit effective immune responses, suggesting that they have evolved alternative immunological strategies for dealing with repeat infections. Moreover, recent genome assembly efforts concomitant with expression analyses have yielded the reconstruction of the evolutionary history of the MHCI (356) and MHCII (357) gene families, demonstrating that teleosts MHC loci have undergone a complex series of gene and genome duplications, culminating in extensive variation in MHC structure and diversity across these animals (358). These distinct teleost species have undoubtedly evolved distinct antigen presentation strategies coinciding with their great diversity across MHCI and II loci. Little is presently know regarding the roles of professional antigen presenting cells such as macrophages in these respective species and it will be most interesting to learn how such cells are integrated within these diverse immune systems.

Distinct fish species also possess several disparate lineages of non-classical MHCs (358), the linkage of which is now believed to have separated before the emergence of tetrapods (359). However, the roles of teleost macrophages and other professional antigen presenting cells in presenting novel antigens in the context of these molecules remain to be explored.

### Teleost DCs

Myeloid-lineage DCs represent heterogeneous populations of professional antigen presenting cells that share a common myeloid progenitor (macrophage-dendritic cell progenitor) with macrophages and are integral to linking the innate and adaptive immune responses (360). Teleosts appear to possess functional analogs to the mammalian DCs and in particular, salmonids have been documented to possessing putative DCs. For example, salmon possess DC-like cells that express MHCII and CD83 (DC marker), are highly phagocytic and exhibit characteristic DC morphology (361). Trout also clearly possess DC-like cells expressing MHCII and other antigen presentation components, many DC markers (362) and exhibiting robust antigen presentation and lymphocyte activation capacities (363). Moreover, trout appear to possess DCs with cross-presentation capacities that express the same hallmark markers seen on the mammalian DCs specialized to antigen cross-presentation (364, 365). Similarly, the cyprinid zebrafish have been shown to possess cells expressing hallmark DC markers and displaying the capacity to present antigens and induce the proliferation of fish CD4^+^ T cells (366).

### The Link Between Teleost Innate and Antibody Responses

It is presently not clear what roles teleost antibodies play in the opsonization of pathogens that enhance macrophage phagocytosis and the canonical Fc receptors responsible for this process in mammals have not been fully elucidated in teleosts (367). However, there are at least five distinct immunoglobulin domain-containing multi-gene receptor families with some structural and signaling motifs seen in the mammalian Fc receptors (368). Moreover, as members of at least one of this family (LITRs) appear to play roles in phagocytosis (369, 370), it is conceivable that members of this, as well as the other receptor families may function as fish phagocytic receptors for antibody-opsonized targets.

While teleost orthologs to the mammalian Fc receptors remain elusive, teleosts are now known to encode poly Ig receptors (pIgRs) that are capable of binding to fish antibodies (371, 372) and appear to be involved in phagocytosis (373) but are not expressed on fish macrophages or B cells (371). It will be interesting to learn whether distinct subsets of fish phagocytes may acquire the expression of pIgRs immune stimuli.

It is notable that cartilaginous fish (sharks) possess IgM-mediated opsonization and cytotoxicity, which is mediated by granulocytes rather than macrophages (374). Turbot macrophage phagocytosis of yeast and beads was greatly enhanced by opsonization with turbot Ig-containing serum fraction however, Ig-opsonized microsporidian spores were not taken up at a greater rate than non-opsonized spores (375). Similarly, brook trout macrophages phagocytosis of *A. salmonicida* was not enhanced when following opsonization of the bacteria by specific fish antibodies although complement-mediated opsonization significantly enhanced bacterial uptake (376). It will be interesting to learn whether the teleost macrophage apparent lack of hallmark Fc receptors reflects in the above observations or whether bony fish macrophages are capable of undergoing antibody-mediated phagocytosis under distinct conditions and through distinct molecular mechanisms.

## Concluding Remarks

Akin to the vast heterogeneity of functionally desperate macrophage subsets observed across mammals, teleost fish appear to possess both a spectrum of functionally distinct macrophage subsets as well as a plethora of potential molecular drivers of these distinct lineages. Moreover and in consideration of the strikingly distinct teleost physiologies, evolutionary and pathogenic pressures as well different repertoires of candidate macrophage differentiation factors, these organisms may well utilize (at least partially) distinct macrophage differentiation and activation strategies. It is notable that while many fish species possess multiple isoforms of key macrophage cytokines, functional studies of these moieties have often been limited to one of the several isoforms and have addressed similarities to the mammalian counterparts whilst overlooking some potential functional differences. Indeed, distinct whole genome duplication events and the ploidy of respective fish species can be seen in disparate cytokine copy-number repertoires amongst even closely related fish species (377). These differences are exemplified in copy numbers of hallmark macrophage cytokines such as IFNγ and TNFα across distinct fish. It is generally assumed that the roles of these respective molecules are conserved to those of mammals. However, it is likely that the retention of multiple isoforms within a particular fish species and the often seen expression differences between these fish cytokine isoforms indicate non-overlapping and possibly novel roles for these respective immune mediators. A greater understanding of the mechanisms of fish macrophage antimicrobial immunity is warranted toward aquacultural applications and for the sake of fundamental research. With greater availability of both fish-specific reagents and genomic resources, the time is ripe for advancing our understanding of these processes.

## Author Contributions

LG, BK, AY, JH, JX, and MB participated in writing the manuscript.

## Conflict of Interest Statement

The authors declare that the research was conducted in the absence of any commercial or financial relationships that could be construed as a potential conflict of interest.
